# Recognizing Relational Interactions with Social Institutions in Refugee Children’s Experiences of Intertwining Vulnerability and Agency

**DOI:** 10.3390/ijerph20196815

**Published:** 2023-09-23

**Authors:** Jeanette A. Lawrence, Agnes E. Dodds, Ida Kaplan, Maria M. Tucci

**Affiliations:** 1 Melbourne School of Psychological Sciences, The University of Melbourne, Melbourne 3010, Australia; agnesed@unimelb.edu.au (A.E.D.); ida@ikaplanpsychology.com.au (I.K.); 2 Victorian Foundation for Survivors of Torture, Brunswick 3056, Australia; tuccim@foundationhouse.org.au

**Keywords:** refugee children, vulnerability, agency interaction co-action, co-construction experiences, refugee situations, recognition specificity

## Abstract

In this paper, we examine relational interactions between refugee children and social institutions, building the case for the recognition of the co-occurrence and intertwining of vulnerability and agency in children’s experiences in diverse refugee situations. This developmental relational approach offers refinement of a general relational worldview by specifying how vulnerable and agentic experiences are co-constructed by children and adult individuals and institutions. We analyze the conceptual roots of vulnerable and agentic experiences, and use the concept of co-construction to specify the processes and outcomes of interactive relational experiences. Evidence from example studies of the intertwining of vulnerability and agency in specific refugee situations demonstrates how refugee children contribute to power-oriented experiences. Due recognition of the relational co-construction of intertwining vulnerable and agentic experiences provides a basis for refining generalized relational observations, and a fine-grained basis for developing policies and procedures to dispel ambivalence to refugee children and to change inequitable policies and practices.

## 1. Introduction

A global trend of ambivalence towards child migrants specifically [1] is fueled by misrepresentations of refugee children as either vulnerable victims totally dependent on host societies, or cunning agents threatening societal stability [2]. Neglect and misrepresentation of these children’s active involvement in social activities and decisions fosters inequity and social exclusion at international and local levels [3].

In a companion paper [4], we investigated how ambivalence towards refugee children disrupts their protection and rights. To address this injustice and its fall-out for children and societies, we argued that a developmental relational approach facilitates repositioning refugee children as rights-bearers and contributors to societal events by identifying the psychological, social, and physical processes and outcomes involved in their relational interactions [5,6,7,8]. The purpose of this paper was to directly apply the general relational approach to two fundamental human experiences that invariably arise in adult–child power imbalances—vulnerability and agency. We propose that children are active contributors to power-oriented interactions in refugee situations. We point to the specific case of how refugee children’s agency co-occurs and intertwines with their vulnerability in interactions with adult institutions. This account of the processes and outcomes of children’s activities in the co-construction and transformation of power structures is a refinement of the general relational understanding of children’s interactions with social insitutions [4]. Concentrated recognition of children’s contributions strengthens the relational basis for working towards their social inclusion and support. According to Taylor’s [9] analysis of social equality, to treat any marginal group with equality and justice involves recognizing, that is, noticing, attending to, and marking, the particular characteristics and actions belonging to their identity, needs, and potential. We add the importance of also marking children’s actions. Focusing on patterns of power relations in refugee children’s interactions with adult authorities provides a basis for fine-tuning policies and practices to foster refugee children’s inclusion and participation in society.

### Theoretical and Methodological Approach

The paper is structured around the argument for recognizing refugee children’s interactions with adult institutions specifically in situations involving power imbalances. By focusing on the co-occurrence and interwining of experiences of vulnerability and agency, we expose the populist ‘vulnerable victim’ motif as a misrepresentation of refugee children’s experiences and capabilities across diverse situations.

The theoretical overview lays out the conceptual roots of vulnerable and agentic experiences that undergird the application of these power-oriented experiences to children, and further undergird a relational approach to adult/child co-constructions of events and beliefs. A power-oriented lens reveals the details of co-occurrences and intertwinings of vulnerable and agentic experiences as refugee children’s contributions to patterns of interaction involving control, dependency, and interdependency, refining a general relational approach. For instance, it can show how children act to either mask or modify their vulnerability and assert some control over events and relationships, and how adult organizations either support or impede children’s actions.

The methodological approach revolves around the conceptual integration and analysis of vulnerable and agentic experiences and their inter-connections. We draw on empirical examples of co-occurrences and inter-connected power dynamics across different refugee situations to support the value of the power-oriented lens. The argument is conceptual, drawing on philosophical accounts of vulnerability and agency and their intertwining that is yet to be foregrounded in intensive empirical work. This is not a review paper and we are wary of the dangers of cherry-picking research evidence. Accordingly, we draw out indications from several studies focused on particular types of refugee situations. Selection was grounded in the material included in the earlier review paper [4], drawing on specific empirical evidence that supports the theoretical proposition of relational connectedness. This method of using empirical examples in support of a theoretical argument is stipulated by the American Psychological Association ([10], p. 8) and is in line with the principles of integration in conceptual papers [11].

This paper is organized to develop the argument. Section 2 presents the fundamental concepts of vulnerability and agency as grounded in philosophical accounts of the relational and situated nature of these power-oriented experiences, with specific application to children. Section 3 then points to supportive examples of the co-occurrences of children’s vulnerable and agentic experiences across diverse refugee situations. Section 4 specifies the co-constructed nature of vulnerability and agency in specific cases. The conclusion points to the importance of recognizing the specificity and contextualization of co-constructed vulnerable and agentic experiences. It also makes a proposal to reframe vulnerability and agency as intertwining experiences that, in some situations, defy separation.

## 2. Theoretical Accounts of Vulnerable and Agentic Experiences

No matter how benign, social institutions organize children’s lives by hierarchically structured adult power. Children relate to such adult authority by their acts of agreement, resistance, or subversion [12,13,14,15]. Vulnerability and agency are foundational relational experiences in children’s engagements with adults, precisely because they involve distributions of power and dependence.

From a relational perspective, an experience is neither exclusively internal nor external: it is contextualized engagement by which internal and external forces interact; *co-act* with each other [6,7]. An environment contributes physical structures, cultural beliefs and practices, and human activities that constrain or enable a person’s experiences, ‘*affording*’ and shaping interactions by the meanings, possibilities, and opportunities they offer or withhold [13,16]. In Dewey’s [17] classic theory of experience, an experience is an interaction: “things interacting in certain ways are experience; they are what is experienced” (p. 5). The person engages with the sociocultural world so that their experience emerges in the process of engaging. The sociocultural environment provides the physical and cultural boundaries and opportunities—affordances—that enable and constrain the person’s activities [13]. The person contributes by giving the environmental material personal meaning as they take in and interpret available affordances in accord with their cultural background, experiential history, self-narratives, and cognitive capabilities [18,19]. The personal contribution may be generated as a pre-engagement intention, or arise once an interaction begins, and become transformed within the dynamic flow of ideas and actions [18,20]. Experiences, then, are made, co-constructed by collaborative internal and external actions. By being made in situ they emerge probabilistically through uncertain processes and produce uncertain outcomes in particular sets of interactions [6]. Consequently, any explanation of an experience that relies exclusively on either personal or social actions is partial and under-determined [21]. If this is so for power-oriented experiences, as we propose, then analyses of refugee children’s vulnerable and agentic experiences cannot be attributed exclusively to either the children or the adult institutions involved.

### 2.1. Experiences of Vulnerability

To be vulnerable is a power-oriented experience involving dependency, powerlessness, and liability of harm [22,23]. People are liable to harm whenever they are open to the environment and particularly to other people who may work from alternative meanings and intentions in their experiential space. In her influential legal theory, Fineman [23] specified that vulnerability is both universal and particular to people’s life circumstances. Because it is universal and inevitable throughout life, she argued, vulnerability is a better foundation for equality than neo-liberal autonomy. Autonomy is experienced by some; vulnerability by all. Whenever a state recognizes that all its people experience vulnerability, it has the responsibility to protect and provide equally for all. Mackenzie [24], applying her relational philosophical perspective, qualified Fineman’s admission of situational variability by claiming that people have vulnerable experiences precisely through their contextualized encounters with others. While people are universally susceptible to vulnerable thoughts and feelings, these are anchored and affected by specific interactions in the phenomenal world.

#### Children’s Experiences of Vulnerability

If all are vulnerable, then official discrimination of some children as ‘not vulnerable’ or ‘more-and-less vulnerable’ requires finer attention to detail than the criteria Derluyn [25] observed in Belgian refugee processing centers. Being vulnerable and thus deserving of inclusion involved being younger than 14 years, female, or having prior refugee status. This kind of bureaucratic categorization of refugee children solely on the basis of narrow categories of vulnerability was not exclusive to Belgium’s migration processes, but is widespread [26]. It ignores how experiences may be modified or buffered as children interact dynamically with specific sets of social forces [27,28]. Abebe, for example, noted how street children in Addis Ababa presented themselves as victims to the public during the daytime but exhibited patterns of unruly agentic behaviour in the streets at night [27].

### 2.2. Experiences of Agency

To be an agent is to be a person who acts, who, according to Buss and Westlund [22], initiates and executes action. Like vulnerability, agency is generalized, personalized, and embodied. It can be expressed in a range of ways in relation to environmental pressures. According to Mackenzie and Stoljar [29], agents are “intrinsically relational because their identities or self-conceptions are constituted by the elements of the social context in which they are imbedded” ([29], p. 22). Agents’ actions cannot be adequately explained without acknowledging their historical and social embeddedness.

Agency is not unfettered self-determination. It is expressed in multiple ways as people engage in the phenomenal world. In philosophical analyses, then, the ‘agency’ concept is generally reserved for human persons as thinking-acting beings who generate intentions, act in the phenomenal world, and reflect on their actions [30]. The ‘autonomy’ concept is specifically reserved for the package of agentic skills and capacities used for controlling one’s own life and for being recognized as a participating agent. Autonomy describes the package of skills and capacities crucial for exerting some control over one’s experiences and for being recognized as a participating agent, for example, by making choices, maneuvering around social rules exploiting loopholes and alternatives. Whenever agents act autonomously, they engage in behaviors or speech for purposes of exerting control over phenomenal happenings. In the social sciences, acts of self-determination are usually described in terms of agency.

#### Children’s Experiences of Agency

Children’s agency tends to be overlooked when it is tied to vulnerability in adult-dominated situations. Kuczynski et al. [14], for instance, noted how, for a hundred years, developmental researchers failed to recognize the implicit agency in young children’s resistance to adult rules and expectations. A twentieth-century paradigm shift, largely driven by the new sociology of childhood, provoked the realization that, like adults, children are active in contributing to the construction and transformation of social phenomena and their own ontology [31,32]. Questions of whether children’s agency involves reduced autonomy may arise because their agency is often generated in circumstances where children are dependent on adults. But the impetus to suggest a lesser form of agency is reduced once the relational nature of all agency is seen as being embedded in networks of relationships and patterns of and openness and interdependence [29,33,34].

In any situation, we may expect to observe a range of interpretations and actions from children as well as from adults as they deal with contextual constraints and possibilities. Some agentic actions by children may be less adaptive and productive than others [27,35]. Gigengack, for example, in a longitudinal follow-up of street children in Mexico discovered a self-destructive paradox of “agency infused with victimhood” ([35], p. 14). Their use of inhalants as a survival mechanism over time turned into self-destruction. Many died in early adulthood due to their earlier agentic organization of their daily survival. Treating the agency of children, like adults, as contributions to co-constructed experiences avoids over-emphasizing the autonomy dimension of agency, but instead acknowledges the contextualization of their thinking-acting contributions. This highlights the possibility of more and less adaptive actions coming from any party in situations where multiple constraints and opportunities are presented in the interactive making of experiences in relationships.

### 2.3. Openness to Vulnerability and Agency

People (adults and children) are not always passive and powerless in the face of threat, and do not always act autonomously. They have adaptive capacity to either serve their own interests or to cope with what they cannot control. The same openness to interpersonal relations that allows people to act autonomously sometimes also threatens them [33]. Engaging with others involves being open to their actions, and that openness simultaneously entails being vulnerable—exposing oneself to the possibility of threat and harm. Avoiding vulnerability may be costly, in light of how it can be intertwined with agency. Anderson made this point strongly in his philosophy of vulnerability [33].

Anderson was concerned with how the same openness to interpersonal relations that allows people to act autonomously sometimes also makes them liable to threat. Vulnerable and agentic experiences are enabled or constrained in the same reflexive loops of personal and social interactions. Autonomous agentic action involves engaging with others. But engaging with others involves being open to their actions as well as to one’s own, and that openness simultaneously entails being vulnerable—exposing oneself to the possibility of harm. Avoiding those situations that carry the potential for making one vulnerable also means closing off the potential for acting autonomously. Shutting down a threatening interaction to avoid vulnerability also risks losing agency.

Accordingly, Petherbridge [34] proposed reframing vulnerability away from negativity towards openness. To be vulnerable then means to have a ‘general openness toward the other’ (p. 591). That openness admits not only the possibility of positive or negative power-related experiences, but also that positive and negative experiences may be so tied up with each other that, in some situations, positive actions may be observed only as they connected with negative actions and vice versa.

Abebe [27] reported research evidence of this kind of openness in street children’s versatile movement from vulnerability to agency, depending on the immediate context. His view of children’s agency resonates with this philosophical theme of openness. He argued that we use a perspective of inter-dependence rather than independence and acknowledge the contextualized fluidty of children’s social agency. We agree.

The idea of openness to experience proposed by Anderson [33] and Petherbridge [34] invites a deep inspection of adult/child interactions; for example, children’s adaptive capacities to influence collective decisions and action has been shown to alter the processes of family therapy [20] and school organization [16]. It also has the potential to institute political change if children’s inclusion is understood in terms of their co-action and is managed with respect instead of tokenism [36].

## 3. Co-Occurrences of Vulnerability and Agency in Refugee Situations

Recognition of children’s active contributions to engagements counters both the entrenched ‘vulnerable victim’ motif of much refugee discourse, and also the unscrutinized and disrespectful reactions to children’s agency that make them more vulnerable, for example, when their inclusion in research and practice decision processes was officially mandated in the UK [36]. Power imbalances abound through all institutional dealings with refugee children, from immigration processing centers to health services and local schools. Official decisions easily slide into inequitable distributions, where children’s vulnerability is made a condition for inclusion and protection [25], and their agentic actions are unrecognized, misrepresented, or trivialized. Dynamic child-by-environment interactions that arise during war, displacement, and resettlement exacerbate pressures on children’s well-being [37] and put them at risk of harm but also expose gaps and loopholes in adult authority that make it possible for them to navigate and exploit to serve their ends.

### 3.1. War and Prolonged Violence

War and prolonged violence often loosen collective practices and conventions that children re-interpret and reconfigure by their activities, although their maneuvers and achievements are often under-reported [38]. For example, Rabaia et al. [39] reviewed 20 studies of violence and displacement for occupied Palestinian communities after the first Intifada (1989–2006). The findings mostly focused on negative associations of children’s exposure to violence (e.g., PTSD, poor psychological adjustment and behavioral symptoms, low self-esteem). Only a quarter paid any attention to positive outcomes (e.g., coping styles, personal growth, social integration). Later researchers [40,41] like Rabaia et al. [39], insisted that the perspectives of Palestinian children must be considered alongside psychiatric diagnosis if we are to understand the heightened power and reduced passivity children experienced along with heightened vulnerability during political struggles. Children’s traumatic experiences of war and violence must be interpreted in light of evidence of their resilience and ingenuity, and their willingness to connect with supportive communities [38].

### 3.2. Refugee Camps

Refugee camps are oppressive environments, but sometimes become places where children exercise unusual forms of control amidst collective vulnerability [42,43]. Where elders abdicate their patriarchal responsibilities, youths step into gaps and previously unimagined leadership roles (e.g., liaising between organizations and community, political activism, family caring) [42]. There are significant mental health consequences for children and family relations in such role reversals and children’s parentification [43].

Most camps are designed to deter self-determination. Paradoxically, Uganda organized its humanitarian support for South Sudanese refugees to reflect the UNHCR [44] policy of self-reliance and resilience. Later, when resources dried-up, it allowed that policy to become a constraint on the efforts of South Sudanese young people. Instead of being a launching pad for their educational and work achievements, the camp became a place of “permanent temporariness” ([45], p. 44). In this case, political policies that should have supported children’s agency became instruments extending their dependency. The intertwining of vulnerability and agency in such cases adds to children’s burdens, promising but not delivering opportunities for self-determination.

Hart’s [46] study of the development of adolescent Palestinian boys in the Hussein camp in Amman demonstrated how different social institutions coalesced with developmental changes as boys developed their identities and aspirations. In their early teen years, they were attached to sport and education and, by around 16 years, prospects for a better life in Jordan outweighed following their elders’ dreams of returning to a poorer Palestine they did not know. Older teenagers refocused their collective identification toward a pan-Islamic community and the vision offered by Islamists. Global opportunities were tied up with localized, Jordanian policies that further marginalized Palestinian refugees and in complex ways, with intergenerational family expectations. Hart argued that young people’s choices cannot be interpreted solely in terms of individualized trauma and vulnerability, but as part of their relation to current inter-generational and political structures.

### 3.3. Resettlement and Recovery

Officials are likely to overlook children’s agency, as O’Higgins [47] observed among social workers who were arranging services for young asylum seekers in the UK. Noting the social workers’ preferences for them to be dependent, the young people quietly made their own arrangements and relationships on the side. Young people resettling in Canada without family support similarly told Denov and Bryan [48] how they navigated through experiences of discrimination, social isolation, and poverty. They described specific strategies they used to redress gaps in the formal support available. A common strategy involved networking although some preferred to retain their privacy. Less common were actions to confront discrimination. Several took police officers to court for racial profiling; others complained to teachers. Many of these young people found Canada, although offering asylum and safety, to be a difficult terrain to interpret and navigate, and elicited multiple and sometimes re-assessed strategies as young people sought support. Denov and Bryan saw the mix of openness and secrecy and trust and mistrust as evidence of the young people’s adaptive agency rather than passivity or pathology.

Interactions between community enabling and children’s personal expressions was at the fore in Daiute’s [49] practice-based studies of narratives adolescents generated in collective workshops. Workshops were conducted in post-war former Yugoslavian and post-massacre Columbian communities. Young people aged between 12 and 27 years displayed relational complexity in the flexible ways they changed their story-telling stances according to their cultural commitments. When telling their experiences of conflict, they aligned their stories with perspective held by their own cultural group, clearly showing they knew “what was okay to say and what is not okay to say” (p. 146). When telling stories about fictional characters, the same adolescents explored contrasting perspectives in their narratives and condemned the whole adult generation without cultural selectivity. The complexity of children’s relational connections included their ability to selectively use collective cultural scripts according to situational demands and the specific purposes of their narratives.

## 4. Co-Constructed Vulnerability and Agency: Processes and Outcomes

A comprehensive review by Arakelyan and Ager [37] found consistent evidence that children’s agency may either buffer or worsen effects of adverse experiences in relation to individual children’s emotionality, coping strategies, adaptability, and attention span. In adversity, children’s agency may only become visible by virtue of its intertwining with vulnerability, for example, when refugee children hide their agency and instead showcase their vulnerability to protect themselves and their families from exposure, social exclusion or enforced return [50,51].

### 4.1. Co-Constructed Manipulations of Power-Oriented Relations

A study by Thompson et al. [52] clearly demonstrates the intertwining of children’s vulnerability and agency in the attempts of unaccompanied minors from Central American countries to enter the USA via Mexico. Thompson et al. deliberately sought to understand how children’s agency was both exercised and thwarted in a range of engagements with adults and institutions. Their in-depth interviews with minors aged between 13 to 18 years also revealed how the children’s agency was inextricably tied to the vulnerability imposed on them by laws and official and unofficial adults who suppressed the young people’s attempts at agentic management of different aspects of their journeys. Adults’ vulnerability-making activities left many children in situations where they were detained without family or consular assistance; forced to sign papers they did not understand; and deceived and threatened by ‘coyotes’ (guides) whom they paid to get them safely across borders. Some children also intentionally retained self-determination through acts of pragmatic dependency by which they surrendered their safety to coyotes or by their strategic parroting of adults ideas and instructions for getting through checkpoints. Some explained how they took calculated risks that suggest a cyclical relationship of vulnerability—leading to—agentic—action—leading to—risk of further vulnerability. Any attempt to separate out the strands misses the tightness of the co-constructions produced by social structures and individualized actions by child and adult participants. For example, Thompson et al. [52] reported the story told by a 15 year-old pregnant girl, Evelin who was too frightened to tell Mexican officials about her agentic flight from her violent boyfriend in Honduras. He had threatened to kill her, her baby, and her family if he were jailed. Too afraid to speak about the potential danger if she were sent back, she witheld information and was sent back. The coalescence of Evelin’s vulnerability in detention, her agentic decision to withold information, and immigration policy put her at risk of further harm. Another form of vulnerability was bound up with the agency by which vulnerable child soldiers and women were able to survive war and social recovery. They ‘self-staged’ their vulnerability and cloaked their agency [51].

Organizational practices may set up and shape children’s vulnerable and agentic experiences without being antagonistic, according to how they allow particular combinations [12]. Huijsmans [53] revealed how institutionalized migration arrangements interacted dynamically with children’s experiences of migrating from Lao villages for work. Children who joined fluid, informal migration networks gained greater scope for exercising agency and managing outcomes than those who joined institutionalized networks. Networks with the assumed safety of contractual relations and legal legitimacy were often organized through village elders. However, the stringent entry requirements reinforced dependence on parents that made children more vulnerable when a migration experience failed. Child migrants were opened to vulnerability or agency by the way their migration networks were organized. Only Huijsmans’ [53] careful attention to the constraints and enablings generated by different networks revealed the effects on children’s experiences.

Co-constructed processes are not available for separate analyses when they are connected in the multiple ways that arise in refugee and migration situations. Vulnerability and agency feed into, modify or sustain each other through different collaborative processes, depending on the cultural and organizational structures prevailing in a particular environment, and through the specific challenges and opportunities that children must navigate. Intergenerational familial expectations and norms, for instance, present different meanings and realities for a young refugee to process than the norms of local migration or camp authorities. Children’s interpretations and acts of independence from family members are likely to differ from those they use to deal with migrant authories [50]. Agentic strands of intertwining experiences may be differentially adaptive and maladaptive as children negotiate and re-negotiate specific vulnerability-making circumstances and responsibilities [27,35]. Accordingy, Abebe [27] argued that children’s agency should be viewed from a perspective of inter-dependence rather than independence. His examples of the relational negotiations of agency in West African rural life illustrates the conceptualization of open relations proposed by Anderson [33] and Petherbridge [34].

### 4.2. Children’s Perspectives on Co-Constructed Power-Oriented Relations

When openness to experience is considered in this relational way, children’s perceptions about their interactions with adults are given appropriate prominence as vital parts of their collaborative experiences. As well as contributing action, they bring their own interpretations to the co-constructed experiences. By way of illustration, we reproduce an unabridged portion of a transcript that illustrates one unaccompanied minor’s perspectives on her resettlement in Australia. Lawrence et al. [54] developed a computer-assisted interview to enable refugee young people to express their thoughts about their wellbeing. The computerized environment gave them personal control over the interview process. Data were complete interview transcripts of four unaccompanied minors that were presented in full comparative tables. This form of interview data allows close textual analysis of the transcript yielding thick evidence of young participants’ perspectives on their interactions with family and authorities. We reproduce the portion of the transcript and make our power-oriented interpretation, with due caution, as illustration of a young person’s view of a co-constructed problem situation.

While the original study was not directly focused on vulnerability and agency, inspection of the transcript yields evidence of 16 year old Asmarina’s (pseudonym) perspectives of her personal contribution to her goal of family reunification in relation to the contributions of others, and especially the migration system. The government’s humanitarian program assigned Asmarina a case worker and access to services assisting family reunifications. Her personalized blending of positive and negative experiences around power and control can be traced through the transcript.

Qn. What do you need to help you feel better?

To feel better I need my family.

Qn: How?

Usually in my mood i just need to be my self and release my anger with my self and try to comfort my self and I pray.

Qn: Is there anybody or anything else that helps you to feel better?

Just God.

When asked about her goals for the next year, she said being re-united with her family would be “my achievement”. She had been separated from them, especially from her mother, for years. She clearly expressed frustration with the limitations of a system that rendered her uncertain and powerless to achieve her goal.

Qn: Is there anything you would like to achieve or change in your life in the next year?

I have application in progerss for my family hole to be succesfull if I see my family that is my achievment.

Qn: Do you think you can achieve that?

Not sure.

Qn: What do you need to help you achieve that?

It is not something under my control.

Qn: Who can help you achieve what you want to achieve?

Case worker, immigration lawyer.

Qn: How?

No answer.

Qn: Is there anything else that would help you achieve?

Am not sure. 

[Transcript reproduced from ([54], Table 3) with Asmirana’s original typed text].

Asmarina grasped the bureaucratic and human constraints on achieving her goal, but she was determined to press forward with a realistic understanding of what she could and could not control. Her perception of the limitations of migration case workers highlights some of the difficulties engaging with the migration system that have resulted in refugee children’s silence and obfuscation in many engagements with officials [50,52]. Her frustration shows a similar depth of understanding of the limitations of specific adults that lay behind the actions of street children in Accra who claimed to have made their own decisions to leave home while also admitting that the adults at home had failed to care for them [55].

In summary, these examples show how children contribute to the co-construction of their experiences of vulnerability and agency by interpretations and actions. Their re-interpretation and manipulation of adult power is normal childhood behavior [15,27,28], and that normality includes refugee children. Abnormality belongs to refugee situations not children [46,56]. Situational abnormality involves institutional failure to provide the resources and support that promote refugee children’s development and social inclusion [16]. As our examples have shown, failure to provide resources and support exist throughout all levels of society, including families and villages, and transitional and resettlement situations [42,46,53,54]. Institutional inadequacies may constrain and shape children’s agency, but they are not independently causal [21]. At all societal levels, refugee children interact relationally with contextualized social forces, working to interpret and reconfigure their own vulnerability by agentically pushing out boundaries of enculturated practices and intergenerational roles [42,46,47,53], manipulating other people’s images of them [51,52], or using institutional systems, concepts, and tools to serve their purposes [40,49,54].

## 5. Conclusions

We began with the value of focusing directly on the co-occurrence and intertwining of vulnerability and agency in children’s experiences in refugee situations. By paying close attention to the specifics of children’s power-oriented interactions with social institutions, developmental relational analyses are able to instantiate general relational concepts of openness and interactivity in situations where children are typically seen as powerless and dependent. Specific situational interactions show how children are not solely and perpetually vulnerable victims in such situations. They are active contributors whose contributions to co-constructed experiences deflect or modify their powerlessness, while also sometimes extending and intensifying their vulnerability [35,52].

Accordingly, we followed Bornstein’s ([5], p. 344) specificity criterion to “mindfully apply” analyses to vulnerable and agentic experiences in specific sociocultural contexts by noticing and marking how children negotiate and renegotiate contextualized power in dynamic collaborations with adult institutions and individuals. The recognition of the co-construction of specific patterns of vulnerability-making and agency-making experiences thus provides a fine-grained basis for dispelling the misconceptions that fuel ambivalence [1,2,4]. Due recognition of the intertwining of children’s vulnerability and agency exposes misconceptions that can be overlooked in abstract accounts [5], and points to concrete, contextualized ways of supporting social inclusion and equity for specific groups of refugee children.

By deliberately focusing on vulnerability and agency and power-oriented co-constructions in specific refugee situations, we were able to examine patterns of child and adult actions that show how specific interconnections of personal and social forces made experiences more or less threatening for children. The abnormal social situations of refugee transitions presented children with a mix of positively and negatively valenced affordances that elicited and supported a range of their positive and negative responses [13,21].

The reframing of vulnerability as openness to both positive and negative aspects of power that Anderson [33] and Petherbridge [34] proposed admits fluid, negotiated, and renegotiated constraining and enabling by personal and social actions. The fluidity of continuing, negotiated social and personal activity in some situations defies separation into vulnerable and agentic strands. Openness to experience may not be in the thinking of resisting children when they re-interpret and reconfigure official procedures and provisions [40,47,54], but by their manipulation of systemic loopholes they contribute to evolving patterns of relieved or intensified vulnerability and expressed or cloaked agency. The intensity of children’s power-oriented interactions with adult authorities varies across contexts, ranging from situations where the constraints and opportunities afforded child workers by migration networks [53] to situations where children deliberately manipulated social imagery by agentic self-presentations of their vulnerability [51,52]. Although framing refugee children’s vulnerability as a perpetual state may permit some nation states to avoid their international obligations [2,3], it is unsustainable in light of the evidence of children’s effective manipulations of varying institutional barriers and loopholes [27,51,52].

Where available, we included the thoughts as well as the actions of refugee children with thick evidence from one study [54]. The form and components of an intertwining vulnerability-making and agency-making experiences depend on situational affordances and the specific psychological, social, and physical processes involved in making relational experiences. For instance, the age-related changes in the identity of Hart’s [46] Palestinian boys cannot be understood apart from the familial, local, and international changes against which those identities were co-constructed. The mix of frustration and determination in Asmarina’s push to achieve family reunification cannot be understood without also understanding the Australian geo-political, bureaucratic, and human limitations on finding and resettling her mother [54]. Child soldiers’ presentations of their vulnerability through agency in victimcy studies [51] is an extreme instance of the kinds of more subtle child manipulations of vulnerability-making situations that cannot be understood without recognizing the dynamic interplay.

Attentive recognition and noticing of possible challenges and opportunities qualifies Fineman’s [23] call to use universal vulnerability as the basis for determining equality in society. Equal distribution of resources also depends on marking and redressing the particular circumstances of the most vulnerable [57]. But vulnerability is not static and not experienced in the same way by all. Understanding the differences in how it is experienced indicates precisely where extra support and provisions are most needed. Just as all refugee children are open to vulnerable experiences by virtue of situated pressures, they also are open to support of their agency. By deliberately focusing on the interwining of vulnerable and agentic co-constructions within refugee situations, we have revealed how different configurations warrant specific attention and remedial action in the service of equality [9,57]. Ambivalent attitudes and unequal provisions may be most appropriately targeted for political and social action to deal with the specific forms of state law or institutional failure that block children’s intrusions into official processes. Recognition of the co-construction of specific actions of vulnerability and agency provides a compelling, fine-grained basis for marking their contributions as natural, present and, in their intertwining, suitable for changing unproductive and inequitable policies and practices.

## Data Availability

No new data were created for this paper.
